# Comparing 1.5 T and 3.0 T MR data for 3D visualization of neurovascular relationships in the posterior fossa

**DOI:** 10.1007/s00701-023-05878-y

**Published:** 2023-11-24

**Authors:** Peter Hastreiter, Olga Maliachova, Rudolf Fahlbusch, Arnd Doerfler, Michael Buchfelder, Ramin Naraghi

**Affiliations:** 1 Department of Neurosurgery, University Hospital Erlangen, Schwabachanlage 6, 91054 Erlangen, Germany; 2 Pediatric Department, Triemli Hospital, Zurich, Switzerland; 3 Clinic for Endocrine Neurosurgery, International Neuroscience Institute, Hanover, Germany; 4 Department of Neuroradiology, University Hospital Erlangen, Erlangen, Germany; 5 Department of Neurosurgery, German Federal Armed Forces Central Hospital Koblenz, Koblenz, Germany

**Keywords:** Neurovascular relationships, Posterior fossa, Compression syndromes, Data fusion, 3D Visualization, 1.5 and 3.0 Tesla

## Abstract

**Background:**

Neurovascular relationships in the posterior fossa are more frequently investigated due to the increasing availability of 3.0 Tesla MRI. For an assessment with 3D visualization, no systematic analyzes are available so far and the question arises as to whether 3.0 Tesla MRI should be given preference over 1.5 Tesla MRI.

**Methods:**

In a prospective study, a series of 25 patients each underwent MRI investigations with 3D-CISS and 3D-TOF at 1.5 and 3.0 Tesla. For both field strengths separately, blood vessel information from the TOF data was fused into the CISS data after segmentation and registration. Four visualizations were created for each field strength, with and without optimization before and after fusion, which were evaluated with a rating system and verified with the intraoperative situation.

**Results:**

When only CISS data was used, nerves and vessels were better visualized at 1.5 Tesla. After fusion, flow and pulsation artifacts were reduced in both cases, missing vessel sections were supplemented at 3.0 Tesla and 3D visualization at 1.5 and 3.0 Tesla led to anatomically comparable results. By subsequent manual correction, the remaining artifacts were further eliminated, with the 3D visualization being significantly better at 3.0 Tesla, since the higher field strength led to sharper contours of small vessel and nerve structures.

**Conclusion:**

3D visualizations at 1.5 Tesla are sufficiently detailed for planning microvascular decompression and can be used without restriction. Fusion further improves the quality of 3D visualization at 3.0 Tesla and enables an even more accurate delineation of cranial nerves and vessels.

## Introduction

The analysis of pathological nerve-vessel contacts in the posterior fossa is crucial for the assessment of neurovascular compression (NVC) syndromes [15, 21]. High-spatial-resolution strong 3D gradient echo T2 weighted imaging, i.e. Constructive Interference in Steady State (CISS) and MR-angiography, i.e. Time-of-Flight (TOF) are established sequences of MR imaging [4, 26, 28].

2D visualization based on slice images is a common approach to assess NVC syndromes. Image data obtained on a MR scanner with 3.0 Tesla (T) field strength has been reported to improve the spatial resolution and hence 2D visualizations compared to image data obtained with 1.5 T field strength [9]. Furthermore, the question arises whether a general recommendation can be made to use only 3.0 T for the evaluation of NVC.

Methods of 3D visualization and image processing have proven to be a powerful support for a more comprehensive spatial analysis of neurovascular relationships [13, 22, 28]. After explicit segmentation of coarse structures, they effectively delineate nerves and vessels with implicit segmentation based on direct volume rendering. With the additional use of a powerful fusion strategy that integrates vascular information from TOF into CISS data, the resulting 3D visualizations are further improved [12]. In this way, problems that limit the quality of 3D visualization such as flow-related signal variability of vessels, pulsation artifacts of the cerebrospinal fluid (CSF) and contour fusion of vessels at the boundary of the CSF are effectively overcome which make the assessment of neurovascular compression more reliable.

A systematic investigation of 3D visualization at 1.5 T compared to 3.0 T seems to be important. Besides examining the 3D visualization of NVC syndromes with 3.0 T data, this work also compares the results with those obtained with 1.5 T data. For both field strengths, it shows the differences of 3D visualizations before and after fusion of CISS and TOF data with respect to the accuracy in reproducing neurovascular details as well as the compensation of imaging artifacts. In this way, this work contributes to a differentiated use of 1.5 T and 3.0 T data.

## Material and methods

### Patients

This study includes 25 patients (f:m, 11:14) with NVC syndromes: Trigeminal neuralgia (TN, *n* = 19), hemifacial spasm (HFS, *n* = 5), and glossopharyngeal neuralgia (GN, *n* = 1). All patients obtained the usual preoperative diagnostic workup and underwent microvascular decompression (MVD). The inclusion criterion was age of 18 years or older. Exclusion criteria were pregnancy and claustrophobia. The ethics committee of the University of Erlangen-Nuremberg approved this study. Informed consent was obtained from all patients included in the study.

### Image data

All patients prospectively obtained image data with MRI at 1.5 T and 3.0 T (Magnetom Sonata and Magnetom Tim Trio, Siemens, Erlangen, Germany) using an institutional protocol to obtain high-resolution 3D-CISS and 3D-TOF data. On both systems, the two sequences were adjusted to isotropic voxel size (0.4 mm) providing equally high resolution in axial, coronal and sagittal orientation (details in [9]). The acquisition of two MRI studies with different field strengths per patient was part of a research initiative.

### Segmentation

The image data was segmented based on a previously presented approach dividing the CISS data into four sub-volumes: (1) CSF area, (2) brainstem, (3) cranial nerves, and (4) all remaining structures [13, 22]. The procedure uses a volume growing strategy to extract the CSF area of the basal cisterns ventral and lateral to the brainstem including all vascular structures. To segment the brainstem, an interactive approach is applied using the segmented CSF region as the ventral boundary and a bounding box as the lateral and dorsal boundaries. Finally, the cranial nerves were separated by manual marking. Volume growing was also used to segment the vascular structures in the TOF data. With regard to the subsequent registration, the segmented vessels in the TOF data were labeled in the same way as the extracted CSF area in the CISS data. Since the quality of the CISS and TOF data can be impaired to varying degrees due to various imaging artifacts, this also has a direct impact on subsequent steps of image processing [9, 12]. In order to improve the segmentation results in these cases, they were additionally processed by manual editing based on expert knowledge in a further step.

### Registration and fusion

To combine vascular information of TOF and CISS data a previously developed registration and fusion strategy was applied, which maps voxels of segmented vessels in TOF data into corresponding voxels in CISS data after their hyper-intense values ​​have been inverted into the hypo-intense value range in CISS [12]. This resulted in a new, fused dataset that can be used to compensate for missing vessels or vessel sections.

### 3D visualization

An implementation of direct volume rendering (dVR) was used for the 3D visualization [13, 22]. It allows a different transfer function to be used for each sub-volume, thereby displaying vessels in red, nerves in yellow and brainstem in light grey. Surrounding structures were rendered completely transparent, as they are irrelevant for NVC syndromes. Four 3D visualizations were produced for each patient for both 1.5 T and 3.0 T, which differ in whether CISS and TOF data were fused or not and to what extent the data were segmented. For an overview, the following terms are used in this work: vis-CISS and vis-FUSION based on CISS data only and fused CISS and TOF data, respectively. When the segmentation was additionally optimized by manual editing based on expert knowledge, the terms vis-CISS-opt and vis-FUSION-opt were used in a similar way.

### Evaluation of results

**Fig. 1:**
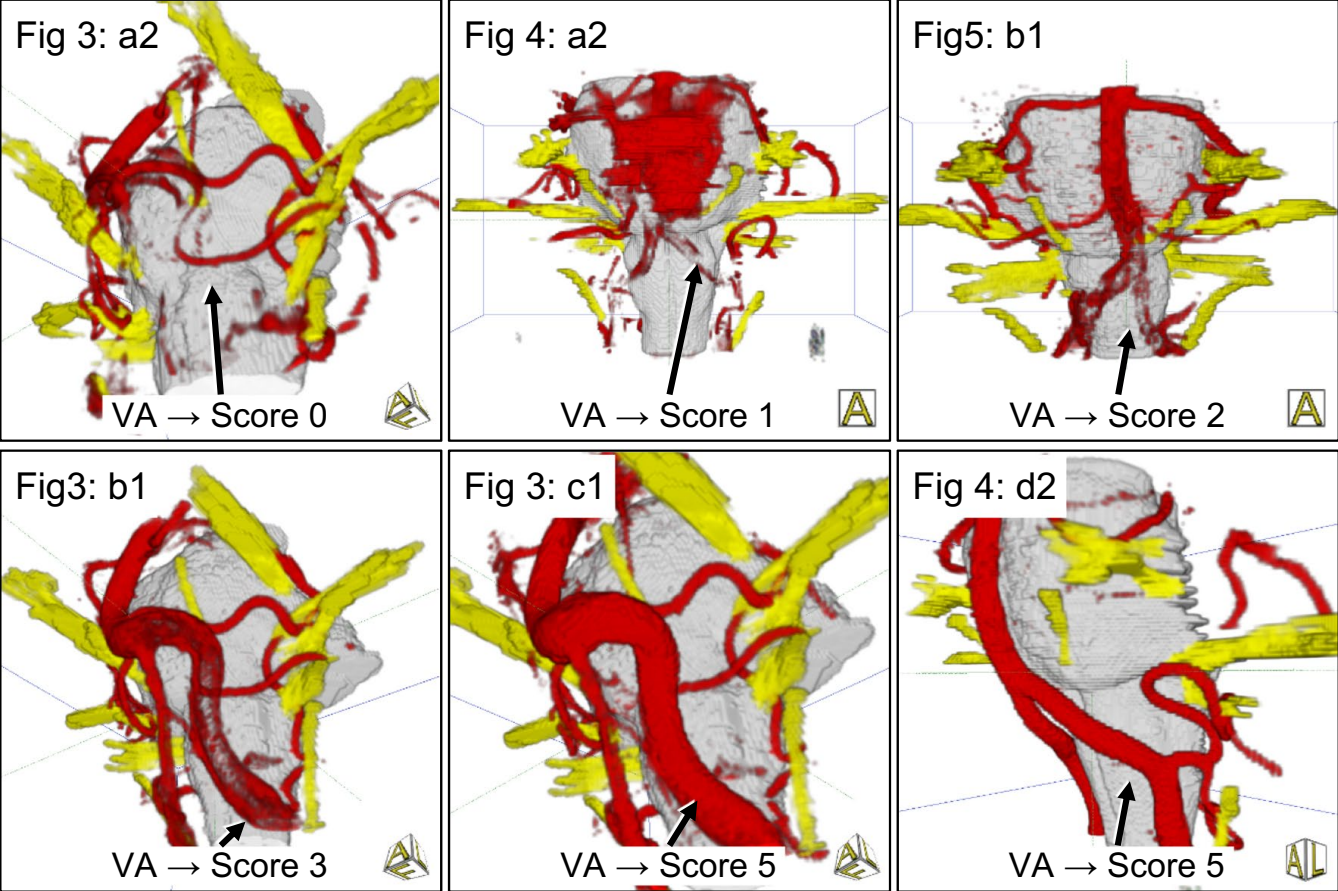
Example of vertebral artery (VA) scoring according to the assessment scheme shown provided in Table 1. The images are taken from the indicated Figs. 3, 4 and 5 according to their image reference

All 3D visualizations were interactively compared and verified with the intraoperative situation during microvascular decompression (MVD). They were evaluated qualitatively and quantitatively. A rating scheme developed by our working group was used to evaluate the quality of the produced visualizations (see Table 1) [12, 19] illustrated in Fig. 1. Two independent expert observers (neurosurgeon with regular experience in MVD) performed the evaluation who were not aware of each other’s assessment. Rating points from 0 to 5 were assigned depending on the presentation of the vessels. Larger and smaller vessels, but also veins close to the brainstem were taken into account. The larger vessels included the basilar artery (BA), the vertebral artery (VA), and the posterior inferior cerebellar artery (PICA), and the small vessels, the anterior inferior cerebellar artery (AICA) and the superior cerebellar artery (SCA). An example of the assigned points is shown in Fig. 1. Another subject of investigation when evaluating the 3D visualizations was the influence of artifacts that arise during imaging [9, 12]. Particular attention was paid to pulsation artifacts caused by CSF pulsation and blood flow, as well as flow artifacts occurring in vessels with a wide lumen.

**Table 1:** The rating scheme for evaluating the 3D visualizations, which was developed by our working group and presented previously [12, 19]

Scoring points	Meaning
0	Vessel not visualized (Representation is completely missing)
1	Vessel poorly shown and can only be guessed (No statements about the exact anatomy are possible)
2	Vessel shown schematically (Peripheral parts recognizable, but important parts are missing)
3	Vessel shown with branching zone (Proximal parts are visible)
4	Vessel shown with relevant parts (Relevant parts related to the compression area are recognizable)
5	Vessel shown completely (Complete representation including all branches)

## Results

The presented approach of 3D visualization was successfully applied in 25 patients suffering from NVC syndromes for preoperative analysis. For the qualitative and quantitative assessment of the results at 1.5 T and 3.0 T, four 3D visualizations each were produced. Every 3D visualization was assessed with the rating system according to Table 1. The mean scores for the respective 3D visualizations are summarized in Tables 2 and 3. Additionally, all 3D visualizations were compared to the intraoperative situation during MVD. All patients tolerated both MR examinations without complications and underwent successful MVD, with no surgical deaths or major complications. The clinical symptoms were resolved in all cases.

**Table 2:** Mean scores (m) for the 3D visualizations using CISS data (vis-CISS) and fused CISS and TOF data (vis-FUSION) at 1.5 T and 3.0 T and distinguishing between left (le) and right (ri) side

Vessels (*n* = 25)	1.5 T	1.5 T	3,0 T	3,0 T
	vis-CISS (m)	vis-FUSION (m)	vis-CISS (m)	vis-FUSION (m)
BA	4,44	4,76	3,84	4,84
VA-ri	3,44	4,68	2,34	4,68
VA-le	3,00	4,68	2,34	4,84
PICA ri	2,12	3,08	0,80	2,76
PICA le	2,68	3,76	1,50	2,88
AICA ri	3,88	4.24	2.96	3,80
AICA le	3,24	3,60	2,57	3,12
SCA ri	4,52	4,68	4,04	4,45
SCA le	4,32	4,66	4,16	4,58

**Fig. 2:**
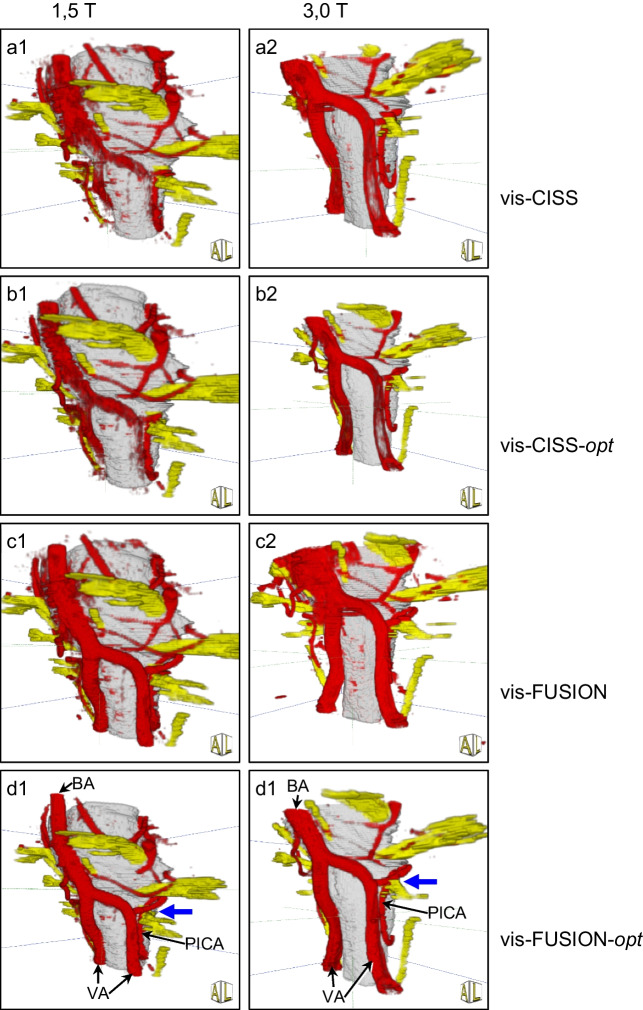
3D visualization of a case with glossopharyngeal neuralgia suggesting NVC by the left PICA (blue arrow) at 1.5 T (a1-d1) and at 3.0 T (a2-d2) demonstrating the larger vessels. Comparing  vis-CISS and vis-FUSION, the positive effect of fusion on the representations of BA, VA and PICA can be seen, which is more pronounced at 3.0 T according to Table 2. The comparison of vis-CISS-opt and vis-FUSION-opt shows the positive influence of fusion on the subsequent optimization, which, according to Table 3, is stronger at 3.0 T

**Fig. 3:**
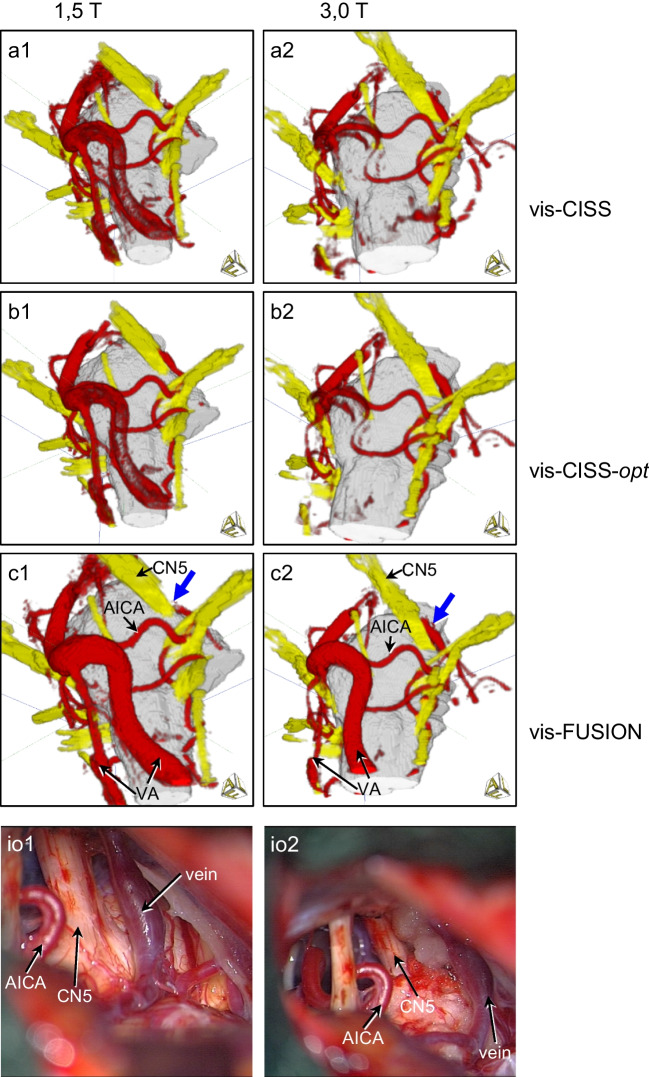
3D visualization of a case with trigeminal neuralgia caused by attachment of a vein on the left side (blue arrow) at 1.5 T (a1-c1) and at 3.0 T (a2-c2) demonstrating the smaller vessels and comparison with the intraoperative situation (io1-2). Note the gap between cranial nerve 5 (CN5) and AICA. The comparison of vis-CISS and vis-FUSION demonstrates the supportive influence of fusion on the representations of AICA and SCA, which, according to Table 2, is comparable at both field strengths, but is smaller compared to the larger vessels. The comparison of vis-CISS and vis-CISS-opt shows an almost unchanged quality of the representations, which is confirmed by only minor improvements in Tables 2 and 3

See Fig. 2 as a demonstration for the larger vessels. Visual demonstrations can be found in Figs. 2 and 3 as well as in Figs. 6 and 7

**Table 3:** Mean scores (m) for the 3D visualizations after optimization with CISS data (vis-CISS-opt) and fused CISS and TOF data (vis-FUSION-opt) at 1.5 T and 3.0 T and distinguishing between left (le) and right (ri) side

Vessels (*n* = 25)	1.5 T	1.5 T	3,0 T	3,0 T
	vis-CISS-*opt* (m)	vis-FUSION-*opt* (m)	vis-CISS-*opt* (m)	vis-FUSION-*opt* (m)
BA	4,60	5,00	4,08	5,00
VA-ri	3,32	4,92	2,25	4,83
VA-le	3,20	4,92	2,25	5,00
PICA ri	2,36	3,00	1,33	3,20
PICA le	2,44	3,28	1,33	3,20
AICA ri	4,12	4,48	3,12	4,33
AICA le	3,32	3,72	2,75	3,70
SCA ri	4,72	4,92	4,47	4,95
SCA le	4,72	4,84	4,60	4,82

Visual demonstrations can be found in Figs. 2 and 3 as well as in Figs. 6 and 7

### Vessel representation

To analyze the obtained scoring results for all 3D visualizations several comparisons were made: In a first study, vis-CISS was compared to vis-FUSION to analyze the impact of fusion with TOF data on the initial CISS data. In another study, Vis-CISS was compared to vis-CISS-opt to see the effect of optimization based on expert knowledge. With the same goal, this comparison was also made between vis-FUSION and vis-FUSION-opt to see how expert-based optimization affects 3D visualization after fusion. In a final study, vis-CISS-opt was compared to vis-FUSION-opt to analyze the impact of fusion in the context of subsequent optimization.

### Impact of fusion on the larger vessels such as BA, VA and PICA

The comparison of vis-CISS and vis-FUSION at 1.5 T shows a maximum improvement of 1.68 for VA-le and a minimum 0.32 for BA (see Table 2). In contrast, at 3.0 T, there is a maximum improvement of 2.5 for VA-le and a minimum of 1.0 for BA. The comparison of vis-CISS-opt and vis-FUSION-opt at 1.5 T leads to a maximum improvement of 1.72 for VA-le and a minimum of 0.4 for BA (see Table 3). In contrast, at 3.0 T, there is a maximum improvement of 2.75 for VA-le and a minimum of 0.92 for BA. The qualitative comparison of 3D visualization before and after fusion confirms the positive impact of fusion on the larger vessels both at 1.5 T and at 3.0 T (see Fig. 2).

### Impact of fusion on the smaller vessels such as AICA and SCA

The comparison of vis-CISS and vis-FUSION at 1.5 T shows a maximum improvement of 0.36 for AICA (le and ri) and a minimum improvement of 0.16 for SCA-ri (see Table 2). In contrast, at 3.0 T, there is a maximum improvement of 0.84 for AICA-ri and a minimum of 0.41 for SCA-ri. The comparison of vis-CISS-opt and vis-FUSION-opt at 1.5 T leads to a maximum improvement of 0.4 for AICA-le and a minimum of 0.12 for SCA-le (see Table 3). In contrast, at 3.0 T, there is a maximum improvement of 1.21 for AICA-ri and a minimum of 0.22 for SCA-le. The qualitative comparison of 3D visualization before and after fusion underlines the impact of fusion also on the smaller vessels both at 1.5 T and at 3.0 T (see Fig. 3).

### Impact of optimization on the vessels

At 1.5 T, the comparisons of vis-CISS and vis-CISS-opt and that of vis-FUSION and vis-FUSION-opt show a maximum improvement of only 0.4 over all vessels (see Tables 2 and 3). For vis-CISS compared to vis-CISS-opt, there is even a slight deterioration of 0.12 for VA-ri and 0.24 for PICA-le. Also vis-FUSION versus vis-FUSION-opt results in a small degradation of 0.08 for PICA-ri and a slightly larger of 0.48 for PICA-le. In contrast, for 3.0 T, vis-CISS versus vis-CISS-opt as well as vis-FUSION versus vis-FUSION-opt leads to a maximum improvement of 0.55 over all vessels (see Tables 2 and 3). Comparing vis-CISS and vis-CISS-opt also results in a slight deterioration of 0.09 for VA (li and re) as well as 0.17 for PICA-le. The comparison of vis-FUSION and vis-FUSION-opt results in only a minimum improvement of 0.16 for VA (le and ri) and BA. The qualitative comparison of 3D visualization with and without optimization, presented in Fig. 2 and Fig. 3, demonstrates that optimization is of secondary importance compared to the effects of fusion.

### Pulsation artifacts

**Fig. 4:**
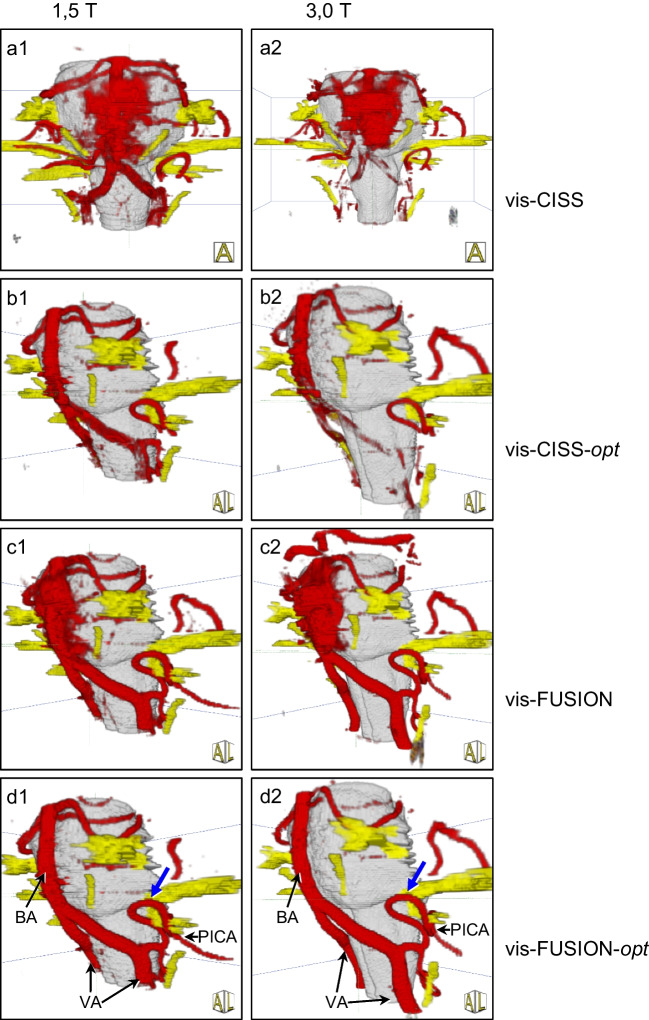
3D visualization of a case with left hemifacial spasm caused by the compression of the PICA to the root exit zone of the nerve (blue arrow) at 1.5 T (a1-c1) and at 3.0 T (a2-c2), showing that optimization reduces pulsation artifacts around the basilar artery (BA). According to Table 4, the comparison of vis-CISS and vis-CISS-opt shows a strong reduction in pulsation artifacts, while only minor improvements can be seen with vis-FUSION. The best reduction is achieved with vis-FUSION-opt, with the representation appearing clearer at 3.0 T, as evidenced by the lower number of artifacts across all vessels in Table 4. The improvements in BA, VA and PICA shown in Tables 2 and 3 also have a positive effect on the representation quality of the vessels

The quantitative analysis of pulsation artifacts is summarized in Table 4. For 1.5 T, optimization with vis-CISS-opt reduces pulsation artifacts by 60–80%. In comparison, using fusion alone with vis-FUSION leads to a reduction of 30–40% on average and 60% in the best case for VA-ri. Furthermore, combined fusion and optimization with vis-FUSION-opt reduces pulsation artifacts by more than 90% in most vessels, with 72% for SCA-ri and 86% for SCA-le being exceptions. For 3.0 T, optimization with vis-CISS-opt significantly reduces pulsation artifacts except for a few vessels. In contrast, using fusion alone with vis-FUSION leads to little or no reduction. Based on the results of vis-CISS-opt, the few remaining pulsation artifacts are further reduced by half with vis-FUSION-opt. Overall, at 3.0 T, minimal optimization was required in only three patients and no optimization at all in one patient. The qualitative comparison of vis-CISS and vis-CISS-opt and that of vis-FUSION and vis-FUSION-opt at 1.5 T and 3.0 T vividly underline the quantitative results (see Fig. 4).

**Table 4:** Number of patients with pulsation artifacts in the examined vessels depending on the type of 3D visualization at 1.5 T and 3.0 T and differentiation between left (le) and right (ri) side

Vessels (*n* = 25)	vis-CISS		vis-CISS-*opt*		vis-FUSION		vis-FUSION-*opt*	
	1,5 T	3.0 T	1,5 T	3.0 T	1,5 T	3.0 T	1,5 T	3.0 T
A. basilaris	14	13	4	2	8	12	1	1
A. vertebralis ri	13	7	5	2	5	5	0	1
A. vertebralis le	12	7	5	2	5	6	0	1
PICA ri	14	4	4	0	11	3	1	0
PICA le	14	5	4	0	12	4	1	0
AICA ri	13	0	2	0	10	0	1	0
AICA le	13	0	2	0	12	0	1	0
SCA ri	7	3	2	1	5	3	2	0
SCA le	14	3	3	1	9	3	2	1

The influence of pulsation artifacts is shown in Fig. 4

### Flow artifacts

**Fig. 5:**
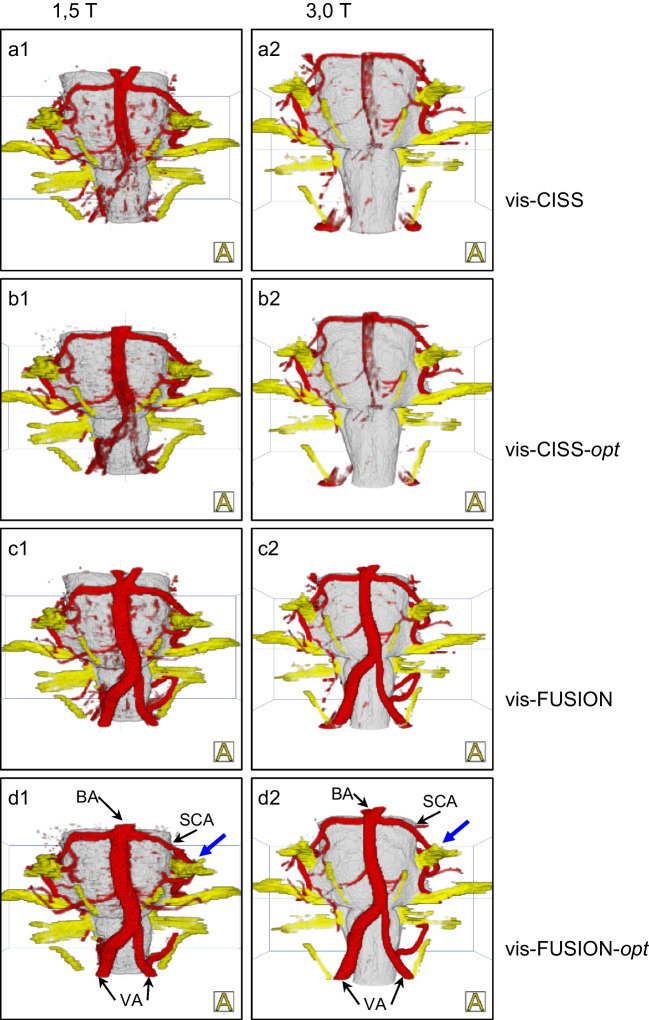
3D visualizations of a case of trigeminal neuralgia caused by NVC by the SCA (blue arrow) at 1.5 T (a1-d1) and at 3.0 T (a2-d2) demonstrating that the flow artifacts are only eliminated after fusion and only then are the basilar artery and both vertebral arteries completely presented. The visual representations are consistent with the quantitative results presented in Table 5

A quantitative assessment of flow artefacts is given in Table 5. For 1.5 T, optimization with vis-CISS-opt leads to no improvement. In contrast, fusion with vis-FUSION results in a reduction of flow artifacts by 90–100%. Subsequent optimization with vis-FUSION-opt does not allow further improvement. For 3.0 T, optimization with vis-CISS-opt leads to almost no improvement either. Here, too, fusion based on vis-FUSION completely eliminates the flow artefacts. A further optimization with vis-FUSION-opt was therefore unnecessary. The qualitative comparison of vis-CISS and vis-FUSION and that of vis-CISS-opt and vis-FUSION-opt at 1.5 T and 3.0 T clearly support the quantitative results (see Fig. 5).

**Table 5:** Number of flow artifacts in the studied vessels depending on the type of 3D visualization at 1.5 T and 3.0 T and differentiation between left (le) and right (ri) side

Vessels (*n* = 25)	vis-CISS		vis-CISS-*opt*		vis-FUSION		vis-FUSION-*opt*	
	1,5 T	3.0 T	1,5 T	3.0 T	1,5 T	3.0 T	1,5 T	3.0 T
A. basilaris	9	16	9	15	0	0	0	0
A. vertebralis ri	19	22	21	21	2	0	2	0
A. vertebralis le	22	23	22	22	1	0	1	0

The influence of flow artifacts is shown in Fig. 5

### 3D visualization at 1.5 T and 3.0 T

**Fig. 6:**
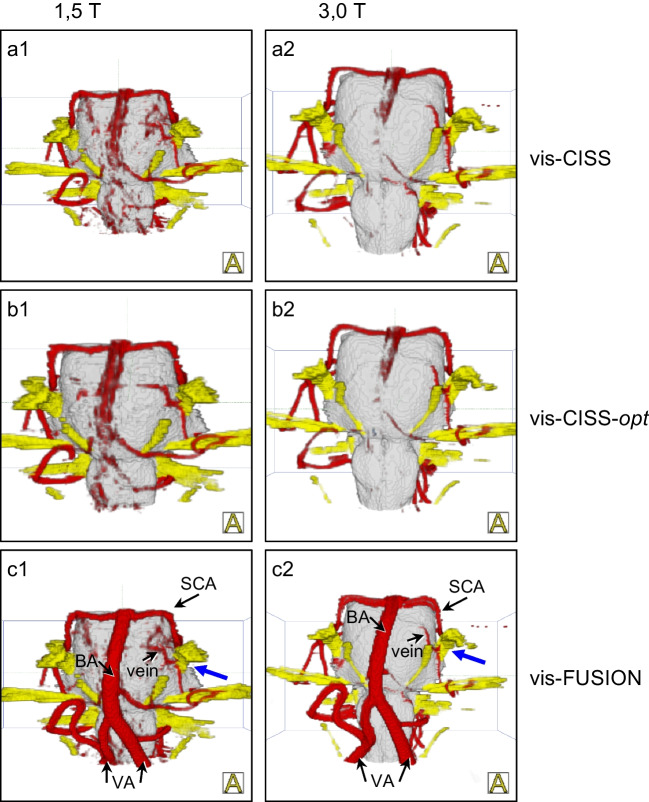
3D visualizations with initially complete absence of the vertebral and basilar arteries and full depiction after fusion in a case of trigeminal neuralgia (blue arrow) caused by NVC of a vein at 1.5 T (a1-c1) and at 3.0 T (a2-c2) – same case as in Fig. 7. As can be seen from Table 2, vis-CISS is better at 1.5 T, especially for the smaller blood vessels, while at 3.0 T many vessels are not displayed at all. Vis-CISS-opt shows a barely noticeable improvement for both field strengths, which corresponds to the small quantitative differences in Table 3. With vis-FUSION, a clear improvement in the representation can be seen for both field strengths in accordance with Table 2, with BA and both VA branches being slightly worse and the smaller vessels being slightly better at 1.5 T

**Fig. 7:**
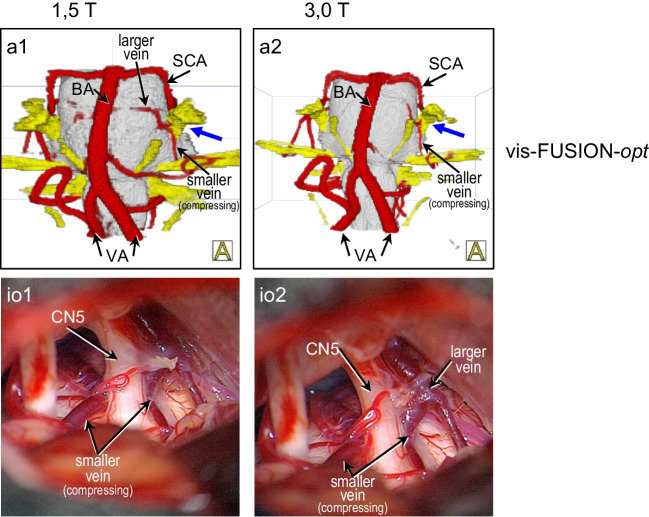
3D visualizations of a case of trigeminal neuralgia caused by a compression of cranial nerve 5 (CN5) by a vein (blue arrow) at 1.5 T (a1) and at 3.0 T (a2) and comparison with the intraoperative situation (io1-2) – same case as in Fig. 6. While the quantitative results in Table 3 show only small differences for all vessels at both field strengths, vis-FUSION-opt at 3.0 T leads to a slightly clearer representation compared to 1.5 T

To compare the results of the 3D visualizations at 1.5 T and 3.0 T, the respective results of vis-CISS, vis-CISS-opt, vis-FUSION and vis-FUSION-opt were examined for each patient. This was achieved both quantitatively using the mean scores of the vessels under consideration (Tables 2 and 3) and qualitatively through visual comparison of the 3D visualizations (see Figs. 6 and 7). The time required for optimization to achieve vis-CISS-opt was up to 3 h for both field strengths, depending on the complexity of the anatomy and artifacts. After fusion, optimization to achieve vis-FUSION-opt took an average of 2 h at 1.5 T and less than 1 h on average at 3.0 T.

### Vis-CISS

Comparing 1.5 T and 3.0 T, the difference in mean values ranges from 0.6 for BA up to 1.32 for PICA-ri, with the visualization at 1.5 T being better by an average of 0.97 for the larger vessels (see Table 2). For the smaller vessels, the difference in mean values is 0.16 for SCA-le and up to 2.0 for AICA-ri, whereby the visualization at 1.5 T was rated better by an average of 0.82 for all smaller vessels (see Table 2). The qualitative analysis of the 3D visualizations showed that many vessels are only very poorly reproduced at 1.5 T and not depicted at all at 3.0 T (see Fig. 6).

### Vis-CISS-opt

Comparing 1.5 T and 3.0 T, there is a difference in mean values of 0.52 for BA and up to 1.1 for PICA-le, with the visualizations at 1.5 T being better by an average of 0.94 for all larger vessels (Table 3). A difference of the mean values of 0.12 is seen for SCA-le and up to 1.0 for AICA-ri with the visualizations at 1.5 T being better by an average of 0.49 for all smaller vessels (Table 3). The qualitative analysis showed that the visualization at 1.5 T provides slightly improved vascular information while many vessels are not displayed at all at 3.0 T either (see Fig. 6).

### Vis-FUSION

Comparing 1.5 T and 3.0 T, the difference in mean values ranges from 0.0 for VA-ri up to 0.88 for PICA-le, with the visualization at 1.5 T being slightly worse for BA and VA-le and slightly better for the other larger vessels (Table 2). For the smaller vessels, the difference in mean values is 0.1 for SCA-le and up to 1.44 for AICA-ri, with the visualizations at 1.5 T being rated better by an average of 0.56 (Table 2). The qualitative analysis showed opposing effects after fusion, which, however, occurred in the same way at both 1.5 T and 3.0 T: While the BA and both VA branches are generally well represented, the root entry/exit zone and the course of the PICA often remain unclear (see Fig. 6).

### Vis-FUSION-opt

Comparing 1.5 T and 3.0 T, there is only a small difference in mean values of 0.1 on average for all vessels (Table 3). Notably, at 1.5 T the mean value is slightly worse for three vessels (VA-le, PICA-ri, SCA-ri) and slightly better for four vessels (VA-ri, PICA-le, AICA-ri, AICA-le) compared to 3.0 T. For two vessels (BA, SCA-le) the mean value was the same at 1.5 T and 3.0 T. The qualitative analysis showed that the visualization is now slightly better at 3.0 T (see Fig. 7), in contrast to the results obtained with vis-CISS, vis-CISS-opt and vis-FUSION.

## Discussion

The ever-increasing availability of MR devices at 1.5 T and 3.0 T also means that examinations of neurovascular relationships in the posterior fossa are being performed more frequently, with compression syndromes and their pathological vascular-nerve contacts being of particular interest. In this context, the presented work shows that MR data at 1.5 T lead to sufficiently detailed 3D visualizations and are sufficient for planning microvascular decompression. In addition, the quality of 3D visualization with MR data at 3.0 Tesla continues to improve and enables even more precise representation of cranial nerves and vessels.

NVC syndromes and their pathological neurovascular contacts have previously been investigated in other studies using various MR imaging protocols. Docampo et al. used 3D steady-state acquisition and 3D-TOF at 3.0 T in TN [6]. Haller et al. saw heavily T2-weighted and angiography sequences as an important basis for identifying the localization of the compressing vessel, whereby no statements were made about the applied field strengths [11]. For the analysis of hemifacial spams, Hermier et al. recommended hyper-T2-weighted sequences and MR angiography at 1.5 T, or even better at 3.0 T, with no further details on the two field strengths given [14]. Xu et al. emphasized increasing magnet strength from 1.5 T to 3.0 T in preoperative assessments of TN, without going into detail about the difference of the two field strengths for the analysis of NVC [29]. Looking at the importance of MR imaging for NVC therapy, Busse et al. identified a greater rate of symptomatic improvement in patients who received preoperative strongly T2-weighted imaging compared to patients without such imaging [4]. Regarding the comparison of 1.5 T and 3.0 T, Garcia et al. reported that the higher signal at 3.0 T together with 2D visualization with slice images allows improved analysis of NVC syndromes [9]. The positive effects of 3D-CISS at 3.0 T for the analysis of NVC were also highlighted by Cavallaro et al. [5].

As the availability of imaging data at 3.0 T has increased, so has the use of 3D visualization for an accurate spatial understanding of the complex relationship of cranial nerves and vascular structures. In this context, the authors contributed an efficient method of 3D visualization [13, 19, 22]. Other contributions correlated the results of imaging and image processing with surgical findings [18, 27] or investigated endoscopic visualization for the intraoperative analysis [8]. Considering veins as another possible cause of NVC, Dumot et al. described the relevance of compression veins as a source of TN [7]. By focusing on the degree of compression in TN based on MR data and MVD findings Sindou et al. [25], Leal et al. [16] and Brinzeu et al. [3] quantitatively investigated its impact on the surgical outcome. In our study, the degree of compression was not evaluated because our investigations focused on the quantitative and qualitative assessment of the 3D visualizations. In particular, typical artifacts of MR imaging were taken into account, which can significantly impair the quality of the 3D visualization or even make an assessment of the NVC impossible. In the future, further research is needed to determine whether the degree of compression of the root can be assessed with the same reliability at 1.5 T and 3.0 T.

In order to obtain a further improved representation of the critical nerve-vessel relationships, the 3D visualization was supplemented by image processing techniques [10, 23, 24, 30]. Since the flow and pulsation artifacts that occur during imaging have a strong influence on the quality of the 3D visualizations, the authors recently introduced an advanced approach of registration and fusion based on high-resolution MR data [12]. In this way, problems limiting the quality of 3D representations such as flow-related signal variability of vessels, pulsation artifacts of the cerebrospinal fluid (CSF) and contour fusion of vessels at the boundary of the CSF are effectively treated.

There have also been studies at 7.0 T to improve the details of the anatomical structures [2], to include tractography [20] or to consider the subnuclei of the limbic structures [1]. However, further research appears to be necessary to allow a comparison with 1.5 T and 3.0 T.

Building on the preceding developments and investigations, this work presents a systematic analysis of 3D visualization of NVC syndromes at 3.0 T compared to 1.5 T. It thus extends the previously discussed results obtained for 2D visualization using slice images only [9]. In contrast to other studies, the presented study focuses on 3D visualization of NVC syndromes and quantitatively and qualitatively investigates the differences between the 3D representations based on the original CISS data without optimization (vis-CISS) and with optimization (vis-CISS-opt) as well as after fusion with TOF data without optimization (vis-FUSION) and with optimization (vis-FUSION-opt). In addition, it addresses how accurately neurovascular details are reproduced and how well imaging artifacts, such as flow and pulsation artifacts, are compensated.

Vis-CISS-opt results in a more noticeable improvement at 1.5 T and a smaller improvement at 3.0 T, which is due to the predominance of pulsation artifacts at 1.5 T and more dominant flow artifacts at 3.0 T in CISS data. Furthermore, vis-FUSION leads to a more moderate improvement in the 3D representation at 1.5 T and a greater improvement at 3.0 T, which is related to the stronger flow artifacts in CISS data at 3.0 T, such that vessel segments are invisible. Finally, vis-FUSION-opt delivers anatomically almost comparably good results at 1.5 T and at 3.0 T, since optimization and fusion complement each other, just in reverse order.

As for pulsation artifacts, these are more pronounced in vis-CISS at 1.5 T than at 3.0 T and they can be successfully eliminated with optimization (vis-CISS-opt) at 1.5 T. However, this is paid for with an increased amount of time for image processing, averaging 1–3 h depending on the level of complexity of the anatomy and the artifacts. At 3.0 T, the optimization of the CISS data is less useful at this point, since the more dominant flow artifacts should be processed first. After fusion, the influence of pulsation artifacts on the 3D visualization (vis-FUSION) is generally weaker, which can be explained by the clearer signal of vascular structures after fusion. Consequently, vessels can then be more easily delineated.

In case of flow artifacts that result in vessel segments being missing in vis-CISS, it is extremely important to supplement the 3D visualization with additional vascular information in order to allow for a reliable assessment of the NVC. Fusion effectively eliminates flow artifacts in the 3D visualization (vis-FUSION) both at 1.5 T and at 3.0 T. At the same time, it should be noted that fusion has a significantly more positive effect on the larger than on the smaller vessels. With subsequent optimization (vis-FUSION-opt), remaining pulsation artifacts could be effectively eliminated both at 1,5 T and at 3.0 T. However, due to the stronger signals at higher field strengths and the positive effects of fusion on the clarity of the vessel representation, which are also more pronounced at higher field strengths, optimization at 3.0 T is easier and more efficient to perform, averaging less than 1 h. In contrast, optimization after fusion at 1.5 T is more complex and takes at least 2 h on average, since the signal differences between the relevant neurovascular structures and remaining artifacts after fusion are smaller.

As a limitation of the MR sequences used in this work, arteries are more reliably depicted than veins. However, it is important to also consider veins as a possible cause of NVC. Even though CISS data can be used to visualize large and small veins, they cannot be distinguished from arteries in the signal. This means that differentiation is not possible without additional expert knowledge, as veins are not well represented with the TOF sequence either. According to Leal et al. the additional use of T1 imaging with gadolinium allowed reliable detection of the NVC and prediction of the extent of the root compression for TN in 1.5 T [17]. Since in our study the MR examinations were performed twice for each patient, gadolinium was given only once for a single T1 sequence. In addition, we also examined other NVC syndromes such as HFS and GN, in which veins are less relevant. Accordingly, the focus of our investigations was on the arterial vascular anatomy and on the quality of its representation when typical artifacts of MR imaging occur. For these reasons, only the CISS and TOF data were analyzed.

In general, 3D visualizations at 3.0 T have higher contrast and sharper contours, which has a positive effect on the reproduction of relevant details and the visual analysis of fine nerve and vessel structures. The vessels, which are often missing in the 3D visualization of the original CISS data at 3.0 T, can be effectively supplemented by fusion, so that remaining artifacts are eliminated with optimization in a more convenient way. Overall, the 3D visualizations at 3.0 T based on fusion and subsequent optimization (vis-FUSION-opt) lead to higher quality results with comparatively less effort for image processing.

## Conclusion

3D visualization is a powerful tool for assessing neurovascular relationships in the posterior fossa. With the work presented, the performance and limitations of 3D visualization at 1.5 T and at 3.0 T in patients with NVC have been systematically analyzed and explained in detail. 3D representations at 1.5 T are sufficiently detailed for planning of MVD and can be used safely. Through fusion and subsequent optimization, the potential of the higher field strength unfolds and 3D visualization at 3.0 T benefits from higher contrast and sharper contours as well as clearer signals after fusion. As this makes planning of MVD more precise, patients with NVC can benefit from the advantages of 3D visualization at 3.0 T.

## Data Availability

The datasets generated and/or analyzed during the current study are available from the corresponding author on reasonable request. All raw data and processed image data are stored on servers of the University Hospital of Erlangen.
